# Celiac artery stenosis threshold for stratifying management in spontaneous isolated dissection

**DOI:** 10.3389/fsurg.2026.1891744

**Published:** 2026-08-26

**Authors:** Taotao Huang, Xiaoting Ye, Tao Zheng, Xiaolong Shu, Zi xuan Wang, Qingyun Zhou

**Affiliations:** 1 Department of Vascular Surgery, Ningbo Medical Center Lihuili Hospital, Zhejiang, China; 2 Department of Nephrology, People’s Hospital of Zhenhai, Ningbo, Zhejiang, China; 3 Department of Vascular Surgery, Zhongshan Hospital, Fudan University, Shanghai, China

**Keywords:** conservative management, endovascular treatment, spontaneous isolated celiac artery dissection, stenosis, vascular remodeling

## Abstract

**Background:**

To identify specific imaging predictors for risk stratification in spontaneous isolated celiac artery dissection (SICAD).

**Methods:**

In this retrospective study, 108 patients with CTA-confirmed SICAD were categorized into Conservative Management (*n* = 57) and Immediate Intervention (*n* = 51) groups. Patients under conservative management were further stratified by celiac artery stenosis severity (≤70% vs. > 70%). Multivariate logistic regression was performed to identify independent predictors of failed vascular remodeling. Primary endpoints included complete remodeling and pain relief.

**Results:**

Patients with celiac artery stenosis >70% under conservative management showed significantly lower complete remodeling (16.7% vs. 56.4%, *P* = 0.012), reduced 3-month false lumen thrombosis (22.2% vs. 66.7%, *P* = 0.005), and longer hospitalization compared to the ≤70% stenosis subgroup. The intervention group demonstrated superior outcomes in true lumen expansion (60.0% vs. 26.4%, *P* < 0.001), complete remodeling (70.6% vs. 43.9%, *P* = 0.013), and shorter hospital stay (3 vs. 6 days, *P* < 0.001). Stent occlusion occurred in 8% of intervened cases, though all remained asymptomatic. Multivariate analysis identified celiac artery stenosis >70% (adjusted OR = 0.24, 95% CI: 0.06–0.90, *P* = 0.035), aneurysm formation (adjusted OR = 0.26, 95% CI: 0.07–0.95, *P* = 0.042), and length of hospital stay (adjusted OR = 0.74, 95% CI: 0.56–0.99, *P* = 0.042) as independent predictors of poor remodeling.

**Conclusion:**

While conservative management is effective for most SICAD patients, celiac artery true lumen significant stenosis as a critical threshold identifying a high-risk subgroup prone to poor remodeling. Clinicians should also integrate the presence of aneurysm formation and a prolonged hospital stay into their prognostic assessments. For patients with this high-risk profile, early endovascular intervention should be considered.

## Introduction

Spontaneous isolated coeliac artery dissection (SICAD) is a rare vascular disorder characterized by dissection of the coeliac artery without involvement of the aorta (1). Although clinically uncommon, its manifestations vary widely, ranging from being asymptomatic and incidentally detected to acute abdominal pain or even severe complications such as visceral ischemia. In recent years, with the widespread use of imaging techniques, the detection rate of SICAD has increased (2, 3). However, there remains a lack of consensus regarding its natural history, treatment strategies, and optimal timing for intervention.

Treatment for SICAD includes conservative and endovascular approaches. Current guidelines from the European Society for Vascular Surgery (ESVS) recommend conservative management for asymptomatic or mildly symptomatic SICAD patients (4). This approach typically involves blood pressure control, analgesia, and occasionally antiplatelet or anticoagulant therapy (5–7).

Ischemia of the pancreas, liver, and stomach may result from a celiac trunk dissection, with subsequent risks of inflammation and infarction (8–10). For patients with persistent symptoms or complications such as aneurysmal expansion or impaired distal perfusion, endovascular intervention or surgical repair should be considered (11–13). Endovascular treatment employs stent placement to reopen the CAD and restore normal blood perfusion to the intestines. Although previous studies have demonstrated the technical feasibility and pain relief, clear imaging or hemodynamic indicators for transitioning from conservative treatment to interventional therapy are still lacking (8, 14, 15).

The hemodynamic consequences of the dissection are largely determined by the degree of compromise of the true lumen. It is hypothesized that a critical degree of stenosis may not only signify a more severe initial injury but also create a hemodynamic environment unfavorable for spontaneous healing, thereby predisposing to disease progression. Kim, B et al. underscores the importance of SICAD morphologic classification in guiding clinical management, suggesting that most types can be managed conservatively, while type IV may require early intervention (16). A study revealed that the presence of false lumen flow in the dissected vessel is a significant predictor of future dilatation in patients with spontaneous isolated dissection of the superior mesenteric artery or celiac artery (17). However, a specific, quantitatively defined stenosis threshold that predicts outcomes has not been established.

This study compares conservative vs. endovascular treatment for SICAD, focusing on how celiac artery stenosis severity affects prognosis. Treatment allocation reflects differences in local interventional expertise and historical practice patterns between two hospital campuses offering real-world evidence. We aim to identify a stenosis threshold that stratifies conservative treatment failure risk, guiding intervention timing and individualized strategies.

## Methods

### Study design

This retrospective study was conducted at the two campuses of XX Hospital, which exhibit differing real-world practices in managing symptomatic SICAD. The choice of hospital was based on patients’ own preference according to residential location and convenience, rather than clinical indication or randomization. Reflecting these practice variations, the study compared two distinct initial treatment strategies employed across the campuses: (1) Initial Conservative Management, and (2) Immediate Intervention. Patients were assigned to one of two groups based on the initial treatment strategy they received.

### Study population

**Figure 1:**
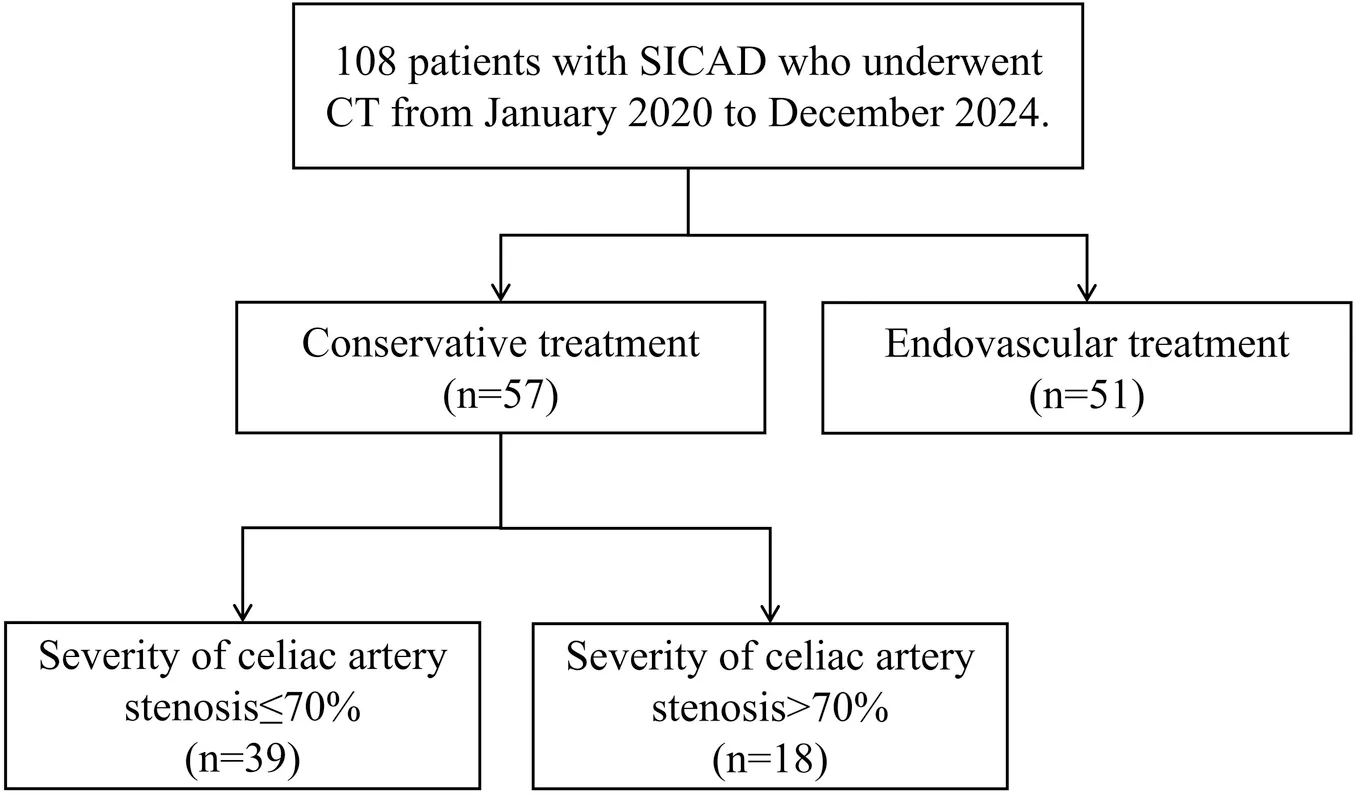
Flow sheet for treatments for SICAD.

We retrospectively analyzed 108 patients admitted to Ningbo Medical Center Lihuili Hospital between January 2020 and December 2024 with a CTA-confirmed diagnosis of SICAD (Figure 1). Patients were excluded if they had dissections involving the celiac artery in conjunction with other visceral arteries, systemic vasculitis, insufficient follow-up data, or had undergone open surgical repair. Based on the initial treatment strategy, patients were categorized into two groups: the Conservative-Initial group (*n* = 57) and the Immediate Intervention group (*n* = 51). Relevant demographic and clinical information, including detailed measurements of celiac artery true lumen stenosis on the initial diagnostic CTA, was extracted from electronic medical records (Table 1). Patients were further stratified into two key subgroups based on baseline celiac artery true lumen stenosis: >70% stenosis (significant stenosis) and ≤70% stenosis. This study was approved by the Ethics Committee of Ningbo Medical Center Lihuili Hospital, adhered to the principles of the Declaration of Helsinki, and was granted a waiver of informed consent.

**Table 1:** Baseline characteristics of patients between the conservative management group and the intervention group.

Variable	ALL (*n* = 108)	Conservative (*n* = 57)	Intervention (*n* = 51)	*P*. overall
Baseline demographics				
Age, years	52.7 ± 10.7	51.2 ± 9.69	54.3 ± 11.6	0.147
Sex, male	96 (88.9%)	52 (91.2%)	44 (86.3%)	0.609
Hypertension	51 (47.2%)	27 (47.4%)	24 (47.1%)	1.000
Hyperlipidemia	39 (36.1%)	22 (38.6%)	17 (33.3%)	0.713
Somking	32 (29.6%)	14 (24.6%)	18 (35.3%)	0.313
Pain score				0.001
1	20 (18.5%)	4 (7.02%)	16 (31.4%)	
2	51 (47.2%)	34 (59.6%)	17 (33.3%)	
3	22 (20.4%)	14 (24.6%)	8 (15.7%)	
4	15 (13.9%)	5 (8.77%)	10 (19.6%)	
Pre-procedural AST, U/L, median [IQR]	23.0 [18.0; 25.0]	22.0 [18.0; 24.0]	23.0 [19.0; 32.0]	0.372
Pre-procedural ALT, U/L, median [IQR]	21.0 [19.0; 28.0]	21.0 [19.0; 29.0]	20.0 [17.0; 24.0]	0.110
Morphological features				
Extent of dissection				<0.001
Only celiac trunk	36 (33.3%)	8 (14.0%)	28 (54.9%)	
Extension to CHA	8 (7.41%)	4 (7.02%)	4 (7.84%)	
Extension to SA	11 (10.2%)	7 (12.3%)	4 (7.84%)	
Extension to LGA	10 (9.26%)	7 (12.3%)	3 (5.88%)	
Extension to CHA and SA	43 (39.8%)	31 (54.4%)	12 (23.5%)	
Splenic infarction	32 (29.6%)	23 (40.4%)	9 (17.6%)	0.018
Aneurysm formation	57 (52.8%)	21 (36.8%)	36 (70.6%)	0.001
Dissection type				0.005
Type I	25 (23.1%)	14 (24.6%)	11 (21.6%)	
Type II	26 (24.1%)	6 (10.5%)	20 (39.2%)	
Type III	30 (27.8%)	20 (35.1%)	10 (19.6%)	
Type IV	27 (25.0%)	17 (29.8%)	10 (19.6%)	
Length of the dissection, mm, median [IQR]	30.0 [16.1; 50.0]	45.0 [23.4; 60.0]	20.0 [12.5; 40.0]	<0.001
Distance from Ostium, mm, median [IQR]	10.0 [6.30; 15.0]	12.0 [8.00; 18.0]	8.94 [6.00; 11.5]	0.004
Residual true lumen percentage (%)	38.3 (16.8%)	40.5 (17.8%)	35.9 (15.4%)	0.158

The mean(SD) deviation was used for normally distributed data, and the median(INR) were used for non-normally distributed variables. IQR, interquartile range. SMA, superior mesenteric artery; CHA: common hepatic artery; SA: splenic artery; LGA: left gastric artery;.

### Strategies for treatment

#### Group 1: initial conservative management

This strategy, implemented primarily at Campus A, involved initial non-operative management. This included blood pressure control, bowel rest (fasting), administration of antiplatelet or anticoagulation therapy (as clinically indicated), and intravenous fluid support. All patients underwent follow-up CTA prior to hospital discharge to evaluate dissection progression. Endovascular intervention [including stent placement [ST] and stent-assisted coiling/ thromboembolization [SACT]], performed by our vascular surgery center, was reserved for cases demonstrating failure of conservative management or clinical deterioration during the admission.

#### Group 2: immediate intervention

This strategy, implemented primarily at Campus B, involved prompt preparation and performance of endovascular intervention upon diagnosis confirmation. Patients in this group proceeded directly to endovascular treatment (ST or SACT) shortly after admission, bypassing a trial of conservative management.

### Image diagnosis and categorization

CTA was performed on a 64-MDCT scanner with tube voltage 100–120 kV, tube current 200 mAs (auto-modulated), 0.625 mm slice thickness, and 0.5 mm reconstruction. Contrast (Iodixanol, 320 mg I/mL) was given at 1.5 mL/kg (4–5 mL/s) Each CTA scan was independently evaluated by a radiology resident in their third year of training, alongside an experienced board-certified radiologist with two decades of specialization in abdominal imaging. Both reviewers collaborated to ensure consensus on the diagnosis through discussion. The imaging features of the coeliac artery dissections were classified according to the Sakamoto classification system (18), which categorizes lesions based on the morphology of the false lumen as follows: Type I: Patent false lumen with both entry and re-entry points; Type II: Cul-de-sac-shaped patent false lumen without a re-entry point; Type III: Thrombosed false lumen with an ulcer-like projection; Type IV: Completely thrombosed false lumen without an ulcer-like projection. The distance from the dissection entry point to the ostium was measured as well. The percentage of celiac artery stenosis was calculated using the following formula: [[1 − (diameter of the true lumen at the narrowest point)/(diameter of the true lumen at the normal distal segment)] × 100%].

### Endovascular therapy

The endovascular procedure was performed by a team of experienced interventional radiologists. The puncture site was usually selected at the femoral artery, and a retrograde puncture was carried out using the modified Seldinger technique. A guiding catheter in conjunction with a guidewire was utilized to super-selectively access the coeliac artery dissection. A stiff guidewire and Fustar adjustable curved sheath were exchanged and advanced to the coeliac artery dissection ostium. The guidewire was navigated into the distal true lumen under fluoroscopic guidance to ensure its position entirely within the true lumen. Subsequently, a self-expanding bare-metal stent (BIOTRONIK Pulsar 18) or covered stent (GORE VIABAHN) was deployed to cover the full length of the dissection. In our center, covered stents were preferred in the majority of cases (42/51, 82.4%) due to their advantage of reliable false lumen exclusion and superior remodeling outcomes. Bare-metal stents were reserved for specific scenario: patients with existing severe splenic infarction, where covered stent placement might potentially exacerbate splenic ischemia. Through a microcatheter (ev3), controlled-release coils or free coils (Boston Scientific) were deployed into the aneurysmal cavity. The contrast agent used during the procedure was Iodixanol.

### Follow-up

All patients underwent follow-up assessments through post-discharge telephone consultations or regular clinical appointments at 1, 3 and 6 months post-treatment, followed by annual evaluations. Additional evaluations were conducted as needed. Both imaging studies and clinical examinations were performed for all patients. Patients with repeated symptoms of severe abdominal pain, abdominal discomfort, or diarrhea were admitted according to CT scan results to find potential underlying issues.

### Definitions

The full healing of the dissection, with no formation of CAD stenosis or thrombus, was referred to as complete remodeling. An enlargement of the true lumen and a decrease in the false lumen were described as partial remodeling. The assessment includes the 3-month false lumen thrombosis, whether the blood vessels have undergone remodeling (including complete remodeling and partial remodeling), and whether the pain has been relieved. Effective conservative care was characterized by alleviation of pain within 2 days, lack of recurrent discomfort, and no progression observed on CTA in subsequent evaluations. Pain was assessed with the numerical rating scale (NRS), a valid and reliable tool for assessing pain. The scale, ranging from 0 (no pain) to 10 (intense pain), classifies pain as mild (1–3), moderate (4–6), and severe (7–10) (19).

### Statistical analyses

Statistical analyses employed IBM SPSS Statistics for Windows, Version 26.0 (IBM Corp, Armonk, New York) and R version 4.1.1 (The R Foundation). Categorical variables were evaluated with Pearson's *χ*^2^ test or Fisher's exact test, while continuous variables were evaluated with the t-test or Mann–Whitney *U* test. Data with normal distribution were shown as mean ± standard deviation, whereas non-normally distributed data were provided as median with interquartile range. Discrimination performance of degree of celiac artery stenosis was assessed by receiver operating characteristic (ROC) curve analysis, and the area under the curve (AUC) was compared using a nonparametric approach. Univariate and multivariate logistic regression analyses were performed exclusively in the conservative management group (*n* = 57) to identify factors associated with vascular remodeling (defined as the presence of remodeling on follow-up imaging). The intervention group was included separately to provide safety and feasibility data for the endovascular approach. Variables with a *P*-value < 0.1 in the univariate analysis were included in the initial multivariate model. Multicategorical variables (dissection extent and dissection type) were incorporated using dummy variables. A backward stepwise selection procedure (with a removal criterion of *P* > 0.05) was used to develop the final multivariate model. Multicollinearity among independent variables in the final multivariable model was assessed using the Variance Inflation Factor (VIF). A VIF value > 10 was considered indicative of significant multicollinearity. Results were presented as odds ratios (ORs) with corresponding 95% confidence intervals (CIs). A *P*-value of below 0.05 denoted statistical significance.

## Results

From January 2020 to December 2024, a total of 108 patients diagnosed with SICAD were enrolled in this retrospective study. Based on the initial treatment strategy, the cohort was categorized into two groups: Conservative Management (*n* = 57) and Immediate Intervention (*n* = 51).

Compared with the conservative management group and the intervention group, there were no significant differences observed regarding baseline demographics. Furthermore, no significant differences were detected in pre- or post-procedural AST or ALT levels between the two main treatment groups (all *P* > 0.05). Significant differences were identified in clinical and morphological features. The intervention group presented with a higher distribution of severe pain scores (*P* = 0.001) and a greater proportion of dissections confined solely to the celiac artery (54.9% vs. 14.0%). In contrast, the conservative group exhibited more extensive involvement including both the hepatic and splenic arteries (54.4% vs. 23.5%), longer dissection length (median 45.0 mm vs. 20.0 mm, *P* < 0.001), and a higher incidence of splenic infarction (40.4% vs. 17.6%, *P* = 0.018). Aneurysm formation was comparable between groups (36.8% vs. 70.6%, *P* = 0.279). The percentage of residual true lumen in the conservative group was higher than that in the intervention group, suggesting that the degree of true lumen stenosis in the conservative group was lower, although there was no statistical difference (Table 1).

Treatment outcomes were notably superior in the intervention group, which demonstrated a greater increase in true lumen diameter (median 60.0% vs. 26.4%, *p* < 0.001), higher rates of complete remodeling (70.6% vs. 43.9%, *P* = 0.013), and a higher incidence of 3-month false lumen thrombosis (82.4% vs. 52.6%, *P* = 0.002). The intervention group also showed a higher rate of pain relief (96.1% vs. 82.5%, *P* = 0.052) and a significantly shorter median hospital stay (3 days vs. 6 days, *P* < 0.001) (Table 2).

**Table 2:** Treatment outcomes between the conservative management group and the intervention group.

Variable	ALL (*n* = 108)	Conservative (*n* = 57)	Intervention (*n* = 51)	*P*.overall
True cavity diameter after treatment	5.95 ± 2.49	5.56 ± 2.64	6.37 ± 2.26	0.088
Percentage of true tone variation	46.5 [19.0; 60.0]	26.4 [0.00; 49.0]	60.0 [46.0; 70.0]	<0.001
Result of vascular remodeling				0.013
Partial remodeling	17 (15.7%)	10 (17.5%)	7 (13.7%)	
Complete remodeling	61 (56.5%)	25 (43.9%)	36 (70.6%)	
No remodeling	30 (27.8%)	22 (38.6%)	8 (15.7%)	
TITFL within 3 months	72 (66.7%)	30 (52.6%)	42 (82.4%)	0.002
Symptom relief	96 (88.9%)	47 (82.5%)	49 (96.1%)	0.052
Post-procedural AST, U/L, median [IQR]	24.0 [20.8; 27.0]	24.0 [22.0; 27.0]	24.0 [19.0; 29.0]	0.946
Post-procedural ALT, U/L, median [IQR]	22.5 [19.0; 25.0]	23.0 [19.0; 25.0]	22.0 [19.0; 25.0]	0.706
Length of hospital stay, days	5.00 [3.00; 7.00]	6.00 [5.00; 8.00]	3.00 [2.00; 4.00]	<0.001

The mean(SD) deviation was used for normally distributed data, and the median(INR) were used for non-normally distributed variables. IQR, interquartile range. TITFL: thrombosis inside the false lumen.

**Figure 2:**
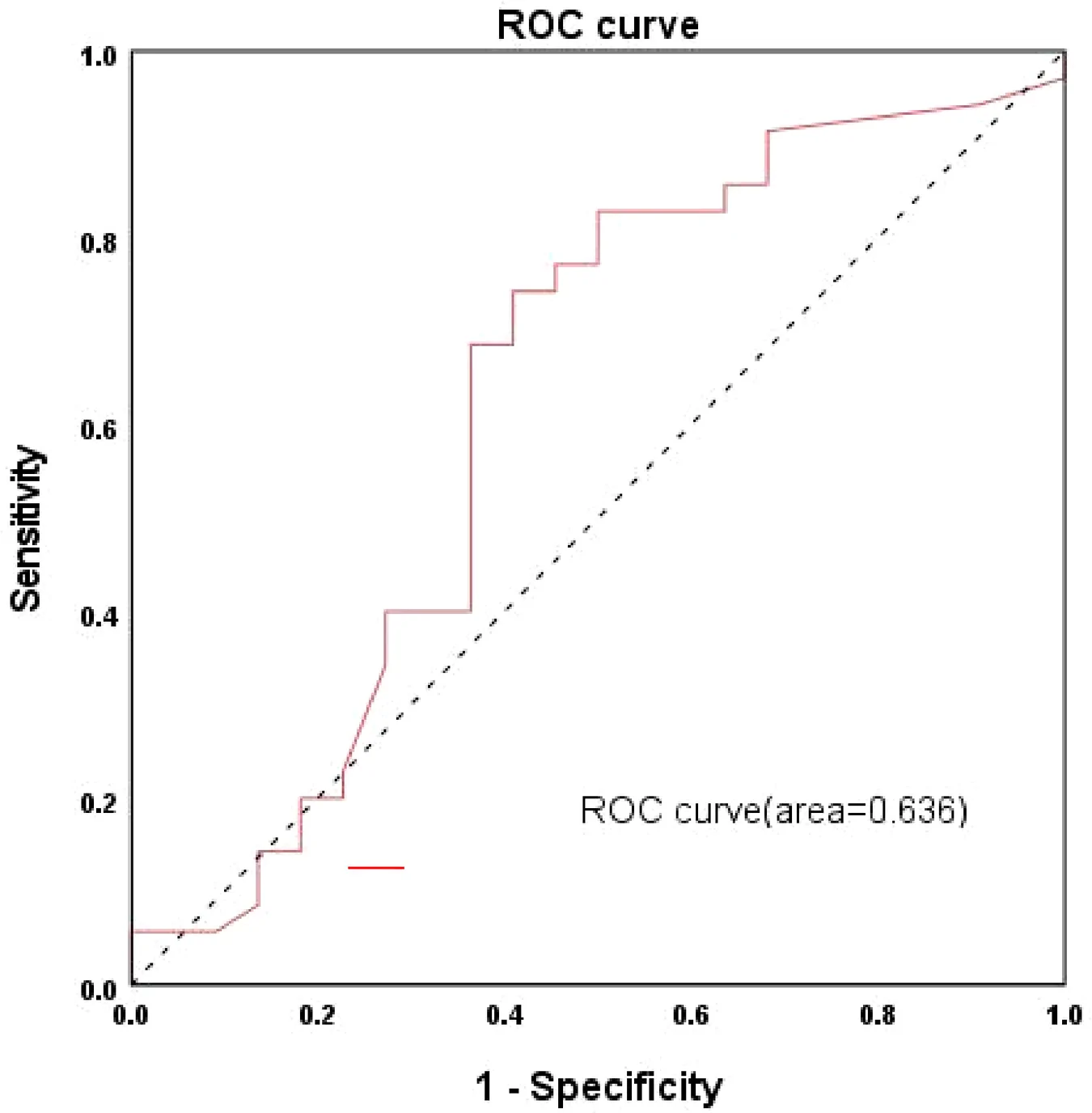
ROC curve for celiac artery stenosis predicting vascular remodeling.

To objectively define the threshold for a severely compromised true lumen, a ROC curve analysis was performed, evaluating the predictive value of the severity of coeliac artery stenosis for the remodeling outcome. The analysis yielded an AUC of 0.636. While the optimal cutoff determined by the Youden index was 65.6% (sensitivity 72.7%, specificity 56.3%), a more conservative threshold of >70% was selected for clinical risk stratification to minimize false negatives and more stringently identify the high-risk subgroup (Figure 2).

In Table 3, a subgroup analysis of patients undergoing conservative treatment was conducted based on the severity of coeliac artery stenosis. Among the 57 patients managed conservatively, stratification based on the severity of celiac artery stenosis (≤70%, *n* = 39; >70%, *n* = 18) revealed no significant differences in baseline demographics. Similarly, pre- and post-procedural AST and ALT levels were comparable between these subgroups (all *P* > 0.05). Morphological severity was also comparable between subgroups, with no significant differences in dissection extent (*P* = 0.927), incidence of splenic infarction (46.2% vs. 27.8%, *P* = 0.306), aneurysm formation (38.5% vs. 33.3%, *P* = 0.938), or dissection length (median 43.4 mm vs. 48.5 mm, *P* = 0.319).

**Table 3:** Baseline characteristics and treatment outcomes in patients undergoing conservative treatment based on the severity of celiac artery stenosis.

Variable	ALL (*n* = 57)	Degree of CAS ≤ 70% (*N* = 39)	Degree of CAS > 70% (*N* = 18)	*P*.overall
Baseline demographics				
Age, years	51.2 ± 9.69	51.1 ± 9.23	51.5 ± 10.9	0.894
Sex, male	52 (91.2%)	34 (87.2%)	18 (100%)	0.168
Hypertension	27 (47.4%)	21 (53.8%)	6 (33.3%)	0.248
Hyperlipidemia	22 (38.6%)	14 (35.9%)	8 (44.4%)	0.746
Somking	14 (24.6%)	11 (28.2%)	3 (16.7%)	0.511
Pain score				0.199
1	4 (7.02%)	2 (5.13%)	2 (11.1%)	
2	34 (59.6%)	26 (66.7%)	8 (44.4%)	
3	14 (24.6%)	7 (17.9%)	7 (38.9%)	
4	5 (8.77%)	4 (10.3%)	1 (5.56%)	
Pre-procedural AST, U/L, median [IQR]	22.0 [18.0; 24.0]	22.0 [18.0; 24.0]	21.5 [18.0; 24.8]	0.877
Pre-procedural ALT, U/L, median [IQR]	21.0 [19.0; 29.0]	22.0 [20.5; 31.0]	20.5 [17.5; 22.0]	0.112
Morphological features				
Extent of dissection				0.927
Only celiac trunk	8 (14.0%)	6 (15.4%)	2 (11.1%)	
Extension to CHA	4 (7.02%)	2 (5.13%)	2 (11.1%)	
Extension to SA	7 (12.3%)	5 (12.8%)	2 (11.1%)	
Extension to LGA	7 (12.3%)	5 (12.8%)	2 (11.1%)	
Extension to CHA and SA	31 (54.4%)	21 (53.8%)	10 (55.6%)	
Splenic infarction	23 (40.4%)	18 (46.2%)	5 (27.8%)	0.306
Aneurysm formation	21 (36.8%)	15 (38.5%)	6 (33.3%)	0.938
Dissection type				0.654
Type I	14 (24.6%)	9 (23.1%)	5 (27.8%)	
Type II	6 (10.5%)	3 (7.69%)	3 (16.7%)	
Type III	20 (35.1%)	14 (35.9%)	6 (33.3%)	
Type IV	17 (29.8%)	13 (33.3%)	4 (22.2%)	
Length of the dissection, mm, median [IQR]	45.0 [23.4; 60.0]	43.4 [23.7; 57.9]	48.5 [21.7; 67.5]	0.319
Distance from Ostium, mm, median [IQR]	12.0 [8.00; 18.0]	12.0 [7.70; 20.0]	10.0 [10.0; 17.1]	0.790
Treatment outcomes				
Percentage of true tone variation	26.4 [0.00; 49.0]	30.0 [5.00; 50.5]	23.2 [−7.50; 30.0]	0.092
Result of vascular remodeling	35 (61.4%)	28 (71.8%)	7 (38.9%)	0.038
TITFL within 3 months	30 (52.6%)	26 (66.7%)	4 (22.2%)	0.005
Symptom relief	47 (82.5%)	34 (87.2%)	13 (72.2%)	0.260
Post-procedural AST, U/L, median [IQR]	24.0 [22.0; 27.0]	24.0 [22.5; 27.0]	23.5 [22.0; 27.0]	0.842
Post-procedural ALT, U/L, median [IQR]	23.0 [19.0; 25.0]	22.0 [19.0; 25.0]	23.0 [21.2; 25.0]	0.848
Length of hospital stay, days	6.00 [5.00; 8.00]	5.00 [4.00; 7.50]	7.00 [6.00; 8.00]	0.038

The mean(SD) deviation was used for normally distributed data, and the median(INR) were used for non-normally distributed variables. Celiac artery stenosis,CAS;TITFL: thrombosis inside the false lumen.

Key outcome differences emerged based on stenosis severity. Patients with greater stenosis (>70%) had significantly worse outcomes. They exhibited lower rates of vascular remodeling (38.9% vs. 71.8%, *P* = 0.038). The high-grade stenosis group also had a significantly lower rate of false lumen thrombosis within 3 months (22.2% vs. 66.7%, *P* = 0.005). There was a trend towards less true lumen expansion in the >70% stenosis group (median 23.2% vs. 30.0%, *P* = 0.092). Pain relief rates were lower in the >70% stenosis group, though not statistically significant (72.2% vs. 87.2%, *P* = 0.260). Reflecting their poorer overall outcomes, patients with high-grade stenosis had a significantly longer median hospitalization duration (7 days vs. 5 days, *P* = 0.038).

To identify factors associated with vascular remodeling, univariate logistic regression analysis was performed exclusively in the conservative management group (*n* = 57). As shown in Table 4, four variables demonstrated statistical significance: celiac artery stenosis > 70% (OR = 0.25, 95% CI: 0.08–0.90, *P* = 0.021), smoking history (OR = 5.22, 95% CI: 1.04–26.17, *P* = 0.045), aneurysm formation (OR = 0.29, 95% CI: 0.09–0.89, *P* = 0.031), and length of hospital stay (OR = 0.69, 95% CI: 0.51–0.92, *P* = 0.012). Other variables, including age, sex, hypertension, hyperlipidemia, pain score, dissection type, dissection extent, dissection length, liver function markers (AST, ALT), and distance from the ostium, showed no significant association (all *P* > 0.05).

**Table 4:** Univariate logistic regression analysis of predictors for vascular remodeling in the conservative management group.

Variable	Odds ratio (95% CI)	*P*-value
Distance from Ostium(per mm)	1.03 (0.98–1.08)	0.306
Degree of celiac artery stenosis > 70%	0.25 (0.08–0.90)	0.021
AST(per U/L)	0.99 (0.96–1.03)	0.551
ALT(per U/L)	0.99 (0.95–1.05)	0.917
Dissection Type		
Type 1	Reference	…
Type 2	0.20 (0.02–2.18)	0.187
Type 3	3.00 (0.7–12.88)	0.139
Type 4	2.40 (0.55–10.53)	0.246
Dissection Extent		
Extension to CHA and SA	Reference	…
Only celiac trunk	0.68 (0.13–3.47)	0.645
Extension to CHA	0.41 (0.05–3.37)	0.406
Extension to SA	0.31 (0.06–1.66)	0.17
Extension to LGA	0.31 (0.06–1.66)	0.17
Age(per year)	0.99 (0.94–1.00)	0.692
Male	0.37 (0.04–3.53)	0.387
Hypertension	2.08 (0.70–6.21)	0.19
Hyperlipidemia	0.63 (0.21–1.87)	0.4
Pain Score(per point)	0.55 (0.26–1.17)	0.123
Smoking History	5.22 (1.04–26.17)	0.045
Splenic Infarction	1.8 1 (0.59–5.51)	0.3
Aneurysm Formation	0.29 (0.09–0.89)	0.031
Length of the dissection(per mm)	0.99 (0.97–1.01)	0.465
Length of hospital stay(per day)	0.69 (0.51–0.92)	0.012

CI, confidence interval; AST, aspartate aminotransferase; ALT, alanine aminotransferase; CHA, common hepatic artery; SA, splenic artery; LGA, left gastric artery.

Variables with a *P*-value < 0.1 in the univariate analysis were subsequently included in a multivariate logistic regression model, Multivariate logistic regression analysis identified celiac artery stenosis > 70% (adjusted OR = 0.24, 95% CI: 0.06–0.90, *P* = 0.035), aneurysm formation (adjusted OR = 0.26, 95% CI: 0.07–0.95, *P* = 0.042), and length of hospital stay (adjusted OR = 0.74, 95% CI: 0.56–0.99, *P* = 0.042) as independent predictors of poor remodeling. Multicollinearity diagnostics showed no significant collinearity (all VIF < 1.25). Using a backward stepwise selection method (with a removal criterion of *P* > 0.05), the final model (Table 5) identified celiac artery stenosis > 70% (adjusted OR = 0.24, 95% CI: 0.06–0.90, *P* = 0.035), aneurysm formation (adjusted OR = 0.26, 95% CI: 0.07–0.95, *P* = 0.042), and length of hospital stay (adjusted OR = 0.74, 95% CI: 0.56–0.99, *P* = 0.042) as independent predictors of vascular remodeling. Specifically, both celiac artery stenosis > 70% and aneurysm formation identified poor vascular remodeling. A longer hospital stay was associated with a lower likelihood of vascular remodeling. Smoking history was not retained in the final multivariate model (*P* > 0.05). The model demonstrated good fit (Hosmer-Lemeshow test, *P* = 0.893) and good discriminatory power (AUC = 0.803).

**Table 5:** Multivariate logistic regression analysis of predictors for vascular remodeling in the conservative management group.

Variable	Adjusted odds ratio (95% CI)	*P*-value
Degree of celiac artery stenosis > 70%	0.24 (0.06–0.90)	0.035
Aneurysm Formation	0.26 (0.07–0.95)	0.042
Length of hospital stay(per day)	0.74 (0.56–0.99)	0.042
Smoking History	…	>0.05

The final model was derived using backward stepwise selection (removal criterion *p* > 0.05). CI, confidence interval.

**Figure 3:**
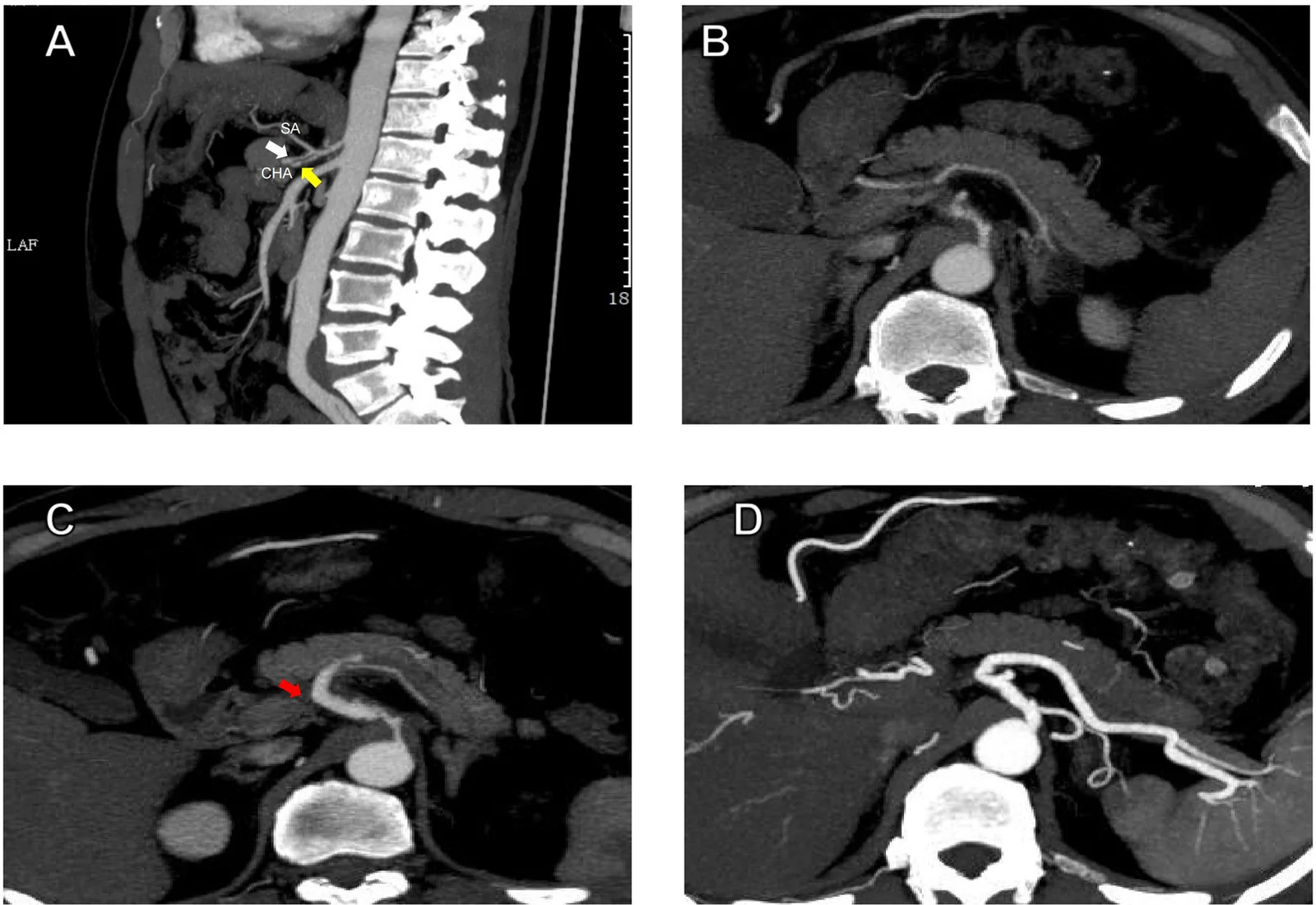
Dissection progression in a conservatively managed patient. **(A/B)** Pretreatment CT [true lumen (white arrowhead), false lumen (yellow arrow)]. **(C)** After 5-day conservative management: non-opacification of proximal hepatic artery (red arrow). **(D)** 6-month follow-up: new collateral circulation in distal hepatic artery. CHA, common hepatic artey; SA, spenic artey.

**Figure 4:**
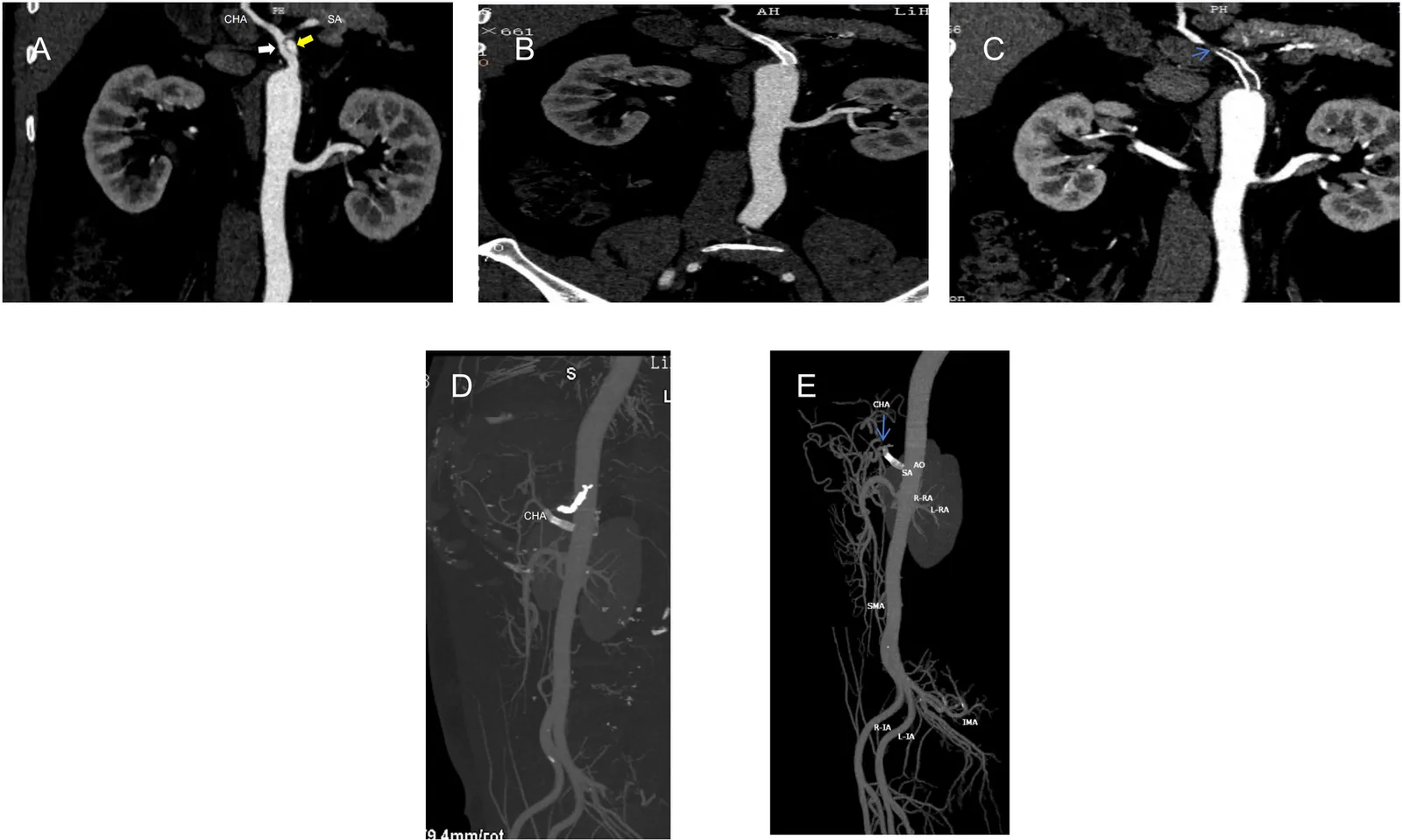
Representative case of stent occlusion after endovascular intervention for spontaneous isolated celiac artery dissection. **(A)** Baseline CTA (CPR) showing true lumen stenosis with false lumen (FL) compressing the true lumen (TL). **(B)** CTA (CPR) at 6-month follow-up demonstrating sustained stent patency. **(C)** CTA (CPR) at 2-year follow-up demonstrating complete stent occlusion (arrowhead). **(D, E)** 3D VR reconstructions at 6-month and 2-year follow-ups. At 6 months **(D)**, the stent is patent with good flow into CHA. At 2 years **(E)**, stent occlusion (arrowhead) with collateral circulation via pancreaticoduodenal arcades maintains visceral perfusion, explaining the asymptomatic status. Mechaism of occlusion: Stent occlusion was likely attributable to incomplete endothelialization of the covered stent, local hemodynamic disturbances, and possible clopidogrel resistance. The patient remained asymptomatic due to robust collateral circulation. AO, aorta; CHA, common hepatic artery; SA, splenic artery; SMA, superior mesenteric artery; TL (white arrowhead), true lumen; FL (yellow arrow), false lumen.

**Figure 5:**
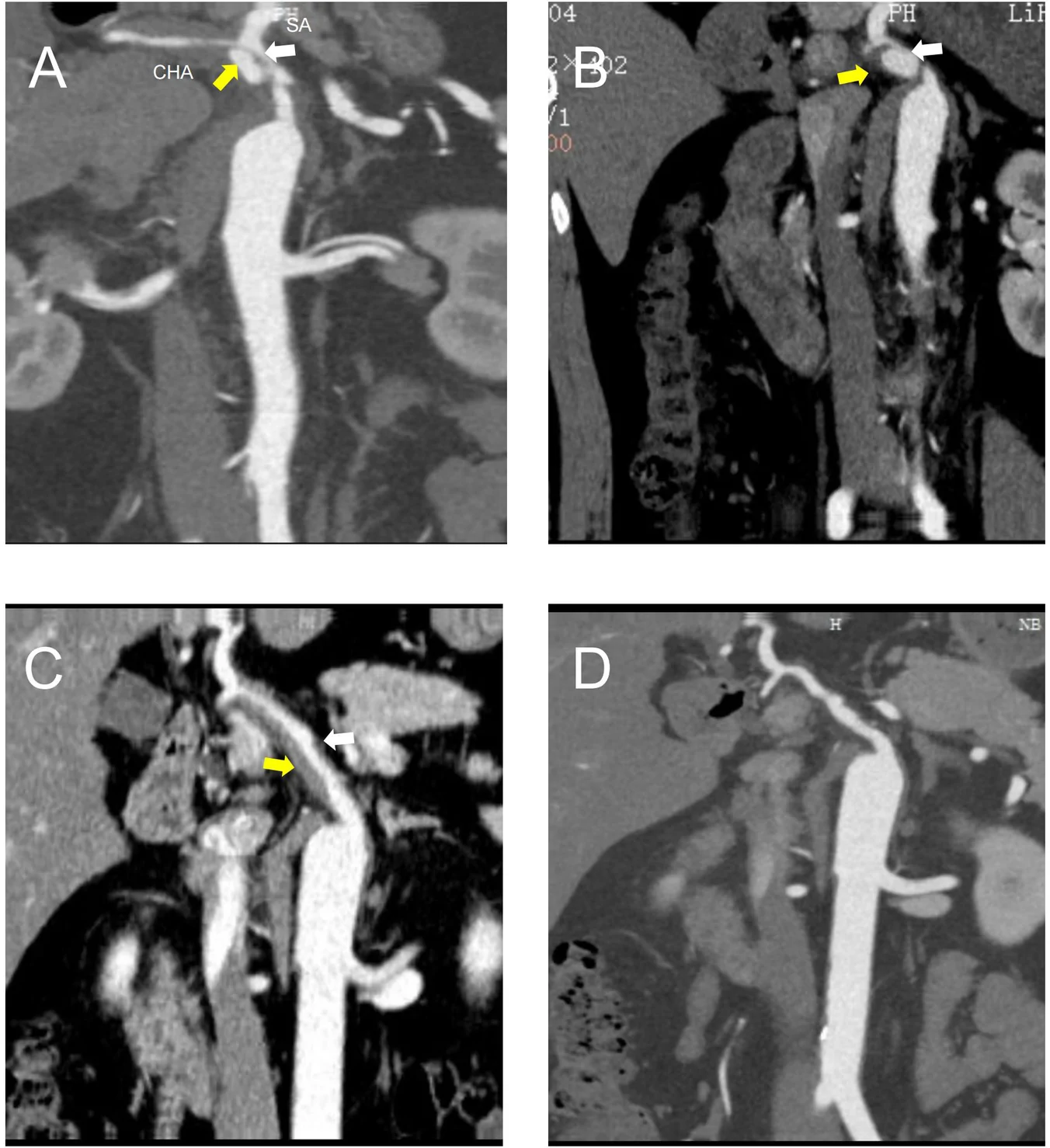
Representative cases illustrating the correlation between baseline stenosis severity and remodeling outcomes at 1-year follow-up. **(A, B)** A patient with >70% stenosis: baseline severe true lumen compromise progressed to false lumen expansion at 1 year, with no remodeling. **(C, D)** A patient with ≤70% stenosis: baseline mild compromise achieved complete remodeling at 1 year with false lumen thrombosis. TL (white arrowhead), true lumen; FL (yellow arrow), false lumen. CHA, common hepatic artery; SA, splenic artery

During the CT follow-up period, in the conservative group of 8 patients (14%), there were cases of progression or occlusion of the dissection (Figure 3). Five patients (8%) chose interventional surgery after the initial conservative treatment failed. And in the surgical group of 4 patients (8%), there were cases of stent occlusion or stent thrombosis during the follow-up period (Figure 4). Additionally, Figure 5 correlates baseline stenosis severity with remodeling outcomes, contrasting a ≤70% stenosis case (complete remodeling) with a >70% case (poor remodeling with persistent false lumen).

## Discussion

SICAD presents a significant clinical challenge due to its rarity and unpredictable natural history. The absence of standardized management protocols often leads to treatment decisions based on individual clinician experience rather than evidence-based criteria. Our study provides important insights into risk stratification and treatment selection for this complex condition.

The findings of our study reaffirm that conservative management remains a viable first-line strategy for a majority of SICAD patients. In our cohort, 82.5% of conservatively managed patients achieved significant symptomatic relief, consistent with previous reports in the literature (20–22). Research by Ethan et al. (12) demonstrated that uncomplicated SICAD can be successfully managed conservatively, with excellent clinical results. Their study noted that although incomplete remodeling and aneurysmal degeneration were common, these findings were nevertheless mostly benign. This success can be largely attributed to the robust collateral circulation network of the celiac axis, which effectively maintains visceral perfusion even in the setting of compromised primary flow (23).

Pain relief was comparable between conservative and intervention groups (82.5% vs. 96.1%, *P* = 0.052), confirming conservative management is effective for most SICAD patients. However, 14% (8/57) of conservatively treated patients showed disease progression or occlusion, underscoring the need for reliable predictors of failure. Additionally, symptom improvement alone is insufficient to guide intervention decisions, as not all patients require surgical treatment. According to Sun et al. (24), endovascular intervention was proposed as a first-line strategy for high-risk cases involving recurrent symptoms, organ malperfusion, or aneurysmal formation. In SIDSMA/SIDCA patients, a study demonstrated a significant association between pseudo-lumen flow and future vessel dilatation, identifying it as an independent predictor (17).

The principal contribution of our study is the identification of >70% celiac artery stenosis as a key imaging threshold that effectively delineates this high-risk subgroup. The superior outcomes observed in the intervention group, including higher rates of complete remodeling (70.6% vs. 43.9%) and true lumen expansion (60.0% vs. 26.4%), provide compelling evidence for early endovascular intervention in selected high-risk patients. Our multivariate regression analysis further elucidated the anatomical determinants of poor remodeling. The degree of celiac artery stenosis > 70% and aneurysm formation as independent predictors provides valuable pathophysiological insights. The significantly reduced odds of successful remodeling associated with these factors (adjusted OR = 0.24 and 0.26, respectively) highlight their clinical importance. The length of hospital stay also demonstrated statistical significance (adjusted OR = 0.74).

The strong association between >70% celiac artery stenosis and unfavorable outcomes can be attributed to its substantial impact on local hemodynamics. Critical luminal narrowing induces abnormal flow velocities and pressure gradients across the lesion, disrupting the physiological conditions required for false lumen thrombosis and vascular wall remodeling. This hemodynamic environment may instead sustain false lumen patency or facilitate its expansion. Moreover, such a high-grade stenosis often indicates more severe initial vessel wall injury and a larger intimal tear, leading to greater compression of the true lumen by the false lumen and consequently impairing the inherent capacity for self-repair.

Similarly, aneurysm formation was strongly correlated with remodeling failure, suggesting more extensive initial damage to the arterial wall. The development of an aneurysm reflects significant disruption of the medial layer, which not only compromises structural integrity but also promotes a hemodynamic milieu that perpetuates false lumen patency or progressive dilatation. Turbulent flow within the aneurysmal sac likely hinders false lumen thrombosis and subsequent healing, thereby reducing the potential for spontaneous vascular repair.

Furthermore, the length of hospital stay was identified as an independent clinical predictor of remodeling outcome. Prolonged hospitalization often serves as a surrogate marker for greater initial clinical severity, persistent symptoms, or complications related to the dissection or its management. Patients experiencing slower recovery or developing complications such as persistent pain or splenic infarction typically require longer inpatient care. This extended clinical course indicates a suboptimal response to initial conservative treatment and is intrinsically associated with a diminished probability of successful vascular remodeling.

It is noteworthy that while stent occlusion occurred in 8% (4/51) of intervened cases, all remained asymptomatic and did not require reintervention. Follow-up CT imaging revealed well-developed peri-stent collateral circulation in these patients, which likely provided adequate compensatory perfusion and explains the absence of ischemic symptoms. This observation underscores the remarkable resilience of the celiac axis's collateral network and reinforces the safety of conservative management for many patients. It also suggests that not all patients who underwent surgical intervention derived a clear benefit.

Stent occlusion or thrombosis is a recognized complication following stent placement. Previous research has suggested that chronic inflammation and neointimal hyperplasia, triggered by mechanical arterial wall injury from the stent, may contribute to this process (25). Other studies have proposed stent length as a potential risk factor for in-stent thrombosis (26). In our cohort, all four cases of stent occlusion occurred in patients receiving covered stents. Although all patients were compliant with dual antiplatelet therapy, the possibility of clopidogrel resistance in some individuals cannot be excluded (27). This observation suggests that future management strategies may benefit from incorporating genetic testing to guide personalized antiplatelet regimens, potentially reducing postoperative adverse events and improving long-term stent patency.

The clinical implications of our findings are substantial. For patients with mild-to-moderate stenosis (≤70%), the preserved true lumen typically maintains accessibility for future interventions if needed. In contrast, patients with >70% stenosis face a substantially higher risk of progression to complete occlusion, which would preclude minimally invasive endovascular approaches and potentially necessitate more complex surgical bypass procedures. This distinction underscores the importance of early intervention in high-risk patients to preserve future treatment options.

This study has several limitations. Its retrospective design introduces the potential for selection bias, as patients chose their treating campus based on geographic convenience, not randomization. The treatment outcomes may have been influenced by inherent institutional preferences. Although the cohort is relatively large for such a rare condition, the sample size still limited the ability to perform more detailed subgroup analyses. Furthermore, the follow-up period, while informative for short- to mid-term outcomes, may not be sufficient to fully evaluate the long-term disease course, such as delayed aneurysm progression in patients managed conservatively. Finally, as the study was conducted within a single tertiary institution, the generalizability of our findings should be validated through future multi-center prospective studies.

## Conclusion

In conclusion, our study advocates for a comprehensive, risk-stratified management algorithm for SICAD. While celiac artery true lumen significant stenosis as a crucial and readily measurable imaging threshold to identify patients at high risk for failed vascular remodeling, clinicians should also integrate the presence of aneurysm formation and a prolonged hospital stay into their prognostic assessments. These factors collectively delineate a high-risk phenotype that may benefit from early endovascular intervention. Such an approach is recommended not only to achieve superior anatomical outcomes but also as a preventive strategy to avert progression to total occlusion, which could compromise future minimally invasive treatment options. For the larger group of patients without these high-risk features, conservative management remains an excellent and effective first-line strategy. The integration of this multi-factor risk profile provides an evidence-based framework to guide clinical decision-making, contributing to more individualized and effective management of SICAD.

## Data Availability

The original contributions presented in the study are included in the article/Supplementary Material, further inquiries can be directed to the corresponding author/s.
